# Identification, characterization and distribution of transposable elements in the flax (*Linum usitatissimum* L.) genome

**DOI:** 10.1186/1471-2164-13-644

**Published:** 2012-11-21

**Authors:** Leonardo Galindo González, Michael K Deyholos

**Affiliations:** 1 Department of Biological Sciences, University of Alberta, Edmonton, AB T6G 2E9, Canada

**Keywords:** Transposable elements, Flax, Genome evolution, LTR elements, Gene expression

## Abstract

**Background:**

Flax (*Linum usitatissimum* L.) is an important crop for the production of bioproducts derived from its seed and stem fiber. Transposable elements (TEs) are widespread in plant genomes and are a key component of their evolution. The availability of a genome assembly of flax (*Linum usitatissimum*) affords new opportunities to explore the diversity of TEs and their relationship to genes and gene expression.

**Results:**

Four *de novo* repeat identification algorithms (PILER, RepeatScout, LTR_finder and LTR_STRUC) were applied to the flax genome assembly. The resulting library of flax repeats was combined with the RepBase *Viridiplantae* division and used with RepeatMasker to identify TEs coverage in the genome. LTR retrotransposons were the most abundant TEs (17.2% genome coverage), followed by Long Interspersed Nuclear Element (LINE) retrotransposons (2.10%) and *Mutator* DNA transposons (1.99%). Comparison of putative flax TEs to flax transcript databases indicated that TEs are not highly expressed in flax. However, the presence of recent insertions, defined by 100% intra-element LTR similarity, provided evidence for recent TE activity. Spatial analysis showed TE-rich regions, gene-rich regions as well as regions with similar genes and TE density. Monte Carlo simulations for the 71 largest scaffolds (≥ 1 Mb each) did not show any regional differences in the frequency of TE overlap with gene coding sequences. However, differences between TE superfamilies were found in their proximity to genes. Genes within TE-rich regions also appeared to have lower transcript expression, based on EST abundance. When LTR elements were compared, *Copia* showed more diversity, recent insertions and conserved domains than the *Gypsy*, demonstrating their importance in genome evolution.

**Conclusions:**

The calculated 23.06% TE coverage of the flax WGS assembly is at the low end of the range of TE coverages reported in other eudicots, although this estimate does not include TEs likely found in unassembled repetitive regions of the genome. Since enrichment for TEs in genomic regions was associated with reduced expression of neighbouring genes, and many members of the *Copia* LTR superfamily are inserted close to coding regions, we suggest *Copia* elements have a greater influence on recent flax genome evolution while *Gypsy* elements have become residual and highly mutated.

## Background

Transposable elements (TEs) influence the evolution, structure, amplification, gene creation, mutation and transcriptional regulation of genes and genomes [1-6]. They are also useful as genetic markers in basic and applied science [7,8]. TEs occupy a substantial fraction of sequenced plant genomes [9], ranging from over 14% in Arabidopsis [10] to more than 80% in maize [11]. Because of their nature and characteristic patterns of insertion [12], TEs may influence large portions of the genome. A study found that one-sixth of all rice genes had some kind of association with TEs [13]. Some TE insertions occur within or near genes, thereby disrupting normal gene expression [12]. Such insertions may influence phenotypic characteristics, as in petal color of gentians [14], or disruption of vitamin E synthesis in sunflower [15]. However, due to gene redundancy or to insertion in regions of the genome that do not affect gene expression, the majority of TE insertions do not have detectable effects on morphology or physiology. For example, neither the insertion of a *Stowaway* element in an intron of the manganese superoxide dismutase gene [16], nor the insertion of retrotransposon *Vine-1* in one member of the alcohol dehydrogenase multigene family [17] affected plant growth and development. Nevertheless, TEs can influence the evolution of plant gene families, as exemplified by disease resistance genes in several plants [18]. Insertions can also result in the capture of gene fragments by TEs, or the adoption of parts of TEs by genes. Some of the clearest examples of gene capture by TEs involve Pack-MULEs. In rice, over 3000 of these gene-carrying transposon-derived elements were found in 440 Mb of sequence [19], and the acquisition of multiple gene fragments from multiple loci may result in the creation of new genes [20]. Genes such as *FAR1* and *FHY3* (involved in the phytochrome signalling pathway), have a conserved transposase-derived region, whose DNA binding and regulatory capacities have been adopted for transcriptional control of downstream genes [21,22]. As was first shown by McClintock in the early experiments that uncovered the *Ac/Ds* TE system in maize [23-26], some types of stress can activate TEs, which can in turn modify gene expression. TE expression triggered by stress has been reported for several elements including: *Tnt1 *[27,28] and *Tto1 *[29,30] in tobacco; *Tos17* in rice [31,32]; and *BARE-1* in barley [33]. However, relatively few active TEs have been identified and several expression studies indicate that transcription and transposition are rare for most elements [12]. While some studies have focused on the expression of individual elements, more recent approaches have compared genome-wide expression data of TEs. These kind of studies have been used to identify TE cassettes in expressed genes in coffee species [34] and Arabidopsis [35], and the activity of different TE families in maize [36] and sugarcane [37]. Flax (*L. usitatissimum*) is one of over 270 species within the family Linaceae, and is a member of the order Malpighiales along with three other species with published whole genome sequences: poplar (*Populus trichocarpa*), cassava (*Manihot esculenta*), and castor (*Ricinus communis*) [38]. Flax is a predominantly self-polinating annual crop grown in temperate regions [39]. Distinct varieties of flax are cultivated for either seed (i.e. linseed) or bast fibers. We recently reported a whole genome shotgun (WGS) assembly of a linseed variety, CDC Bethune [40]. The assembly contains 302Mb of the estimated 373Mb nuclear genome, in scaffolds with N_50_=694kb. Flax is considered a diploid (2n=2x=30), although our genome analysis pointed to a recent whole genome duplication 5-9MYa. Flax appears to have originated from its wild relative, *L. bienne*, with cultivation and domestication probably starting in the Mesopotamian valleys between 8000–10000 years ago [41]. Flax has been studied for decades as a model of genome plasticity [42-45]. In the variety Stormont Cirrus, individuals exposed to certain stresses can produce first generation progeny that show stable changes in several traits including an up to 15% difference in nuclear DNA content. Highly repetitive, tandemly arrayed elements (e.g. 5S rDNA) are among the major contributors to this DNA content variation. A novel, non-TE, low-copy insertion sequence (LIS-1) is also associated with these changes [42,46]. It should be noted that most elite flax varieties, including CDC Bethune, which is the subject of the WGS assembly, do not exhibit this rapid change in genome size. Nevertheless, the study of flax and its repetitive sequences remains of special relevance to understanding genome evolution in general. We previously reported the preliminary identification of TEs as part of the description of the flax WGS assembly [40]. The assembly contained 23.06% TEs as defined by sequence coverage. While the calculated proportion of the genome covered by TEs in flax is slightly lower than other plant species with small genomes, much variation exists in TE content in plants [9]. Only a small proportion of the TEs described in the flax genome could be identified through alignment to previously characterized elements from other species [40]. Instead, most of the TEs were identified only by *de novo* prediction methods. Here we extend this previous report to present a detailed characterization of the main superfamilies of TEs in flax and to explore their potential influence on genome evolution and gene expression.

## Results

### TEs in the flax genome

In a previous study, we described a *de novo* whole genome shotgun (WGS) assembly of flax based on next-generation (Illumina) sequencing [40], including a brief description of the transposable element (TE) component of that assembly. Using various bioinformatics tools to identify repeats *de novo*, we found a total of 8,162 putative interspersed repeats divided into 456 consensus interspersed repeats for PILER, 5,440 repeats for RepeatScout, 1,977 LTR elements from LTR_finder and 289 LTR elements using LTR_STRUC (Additional file 1). Each of these tools offered certain advantages. For example, PILER was faster and had longer average output sequence length (882.5 bp) than RepeatScout (353.5 bp), but at the same time PILER was more stringent and identified fewer sequences. Furthermore, LTR_finder found more sequences than LTR_STRUC but a few sequences were only found using LTR_STRUC. Although the parameters used with the algorithms for *de novo* repeat finding were set to find interspersed repeats and to filter out low complexity regions, some of the repeats identified may have nevertheless constituted non-TE gene families, pseudogenes or highly repeated gene domains. We therefore curated the repeats to identify those that most likely represented TEs. After curation, the filtered library had a total of 2142 putative TEs: 85 from PILER, 767 from RepeatScout, 1039 from LTR_finder and 251 from LTR_STRUC (Additional file 1). We combined these annotated *de novo* repeats with the TEs from the *Viridiplantae* division of Repbase, to make a database for Repeatmasker, which, when applied to the flax genome assembly, masked a total of 73.8 Mb (23.06% of the assembly) as sequence with high similarity to TEs (Table 1). LTR retrotransposons of the superfamilies *Copia* and *Gypsy* were the dominant group with over 69% of the hits and over 74% of the sequence coverage. These superfamilies were followed by the non-LTR retrotransposons of the L1 group and the DNA transposons from the *Mutator* superfamily. When these results were compared with the analysis of 54.6 Mb of Sanger dideoxy sequence reported for BAC-ends from the same flax variety [47], we found that both data sets showed LTR elements to be the most prevalent group of TEs, with the *Copia* group as the most abundant type followed by *Gypsy* elements and LINEs. However, *hAT* elements were more abundant than *Mutator* elements in the BAC-end sequences, in contrast to our observations from the WGS assembly. Other smaller groups also differed in their rank order in the BAC and WGS analyses, and the total proportional coverage for the WGS was always higher than for the BAC-end sequencing. This was probably due to differences in methodology; whereas in the present study we used both similarity-based and *de novo* identification, the BAC-end analysis relied mainly on similarity-based approaches for repeat identification. 

**Table 1:** **Annotation of TE superfamilies in flax WGS assembly determined using a filtered consolidated library produced with *de-novo *repeats from PILER, RepeatScout, LTR_finder and LTR_STRUC, and a library of TE from the *viridiplantae *division from Repbase**

**Class**	**Order**	**Superfamily**	**No. of matches**	**Elements percentage (%)**	**Sequence occupied (bp)**	**Sequence percentage of TEs (%)**	**Sequence percentage of genome (%)**
**Retrotransposons**	**LTR**	***Copia***	89951	38.31	29594882	40.08	9.30
		***Gypsy***	72626	30.93	25123127	34.02	7.89
		***unclassified***	2797	1.19	902298	1.22	0.28
	**DIRS**	***DIRS***	2	0.00	102	0.00	0.00
	**PLE**	***Penelope***	548	0.23	30214	0.04	0.01
	**LINE**	***RTE***	11	0.00	618	0.00	0.00
		***L1***	27632	11.77	6684243	9.05	2.10
	**SINE**	***unclassified***	1	0.00	49	0.00	0.00
**DNA transposons**	**TIR**	***Tc1-Mariner***	191	0.08	38231	0.05	0.01
		***hAT***	7935	3.38	1986522	2.69	0.62
		***Mutator***	21124	9.00	6320424	8.56	1.99
		***P***	2	0.00	96	0.00	0.00
		***Harbinger***	1384	0.59	344876	0.47	0.11
		***En-Spm/CACTA***	8330	3.55	2372592	3.21	0.75
	**Helitron**	***Helitron***	2154	0.92	434859	0.59	0.14
	**unclassified**	***unclassified***	95	0.04	8981	0.01	0.00
**TOTALS**			**234783**	**100.00**	**73842114**	**100.00**	**23.06**

### Putative expression and abundance of main families of TEs

**Figure 1:**
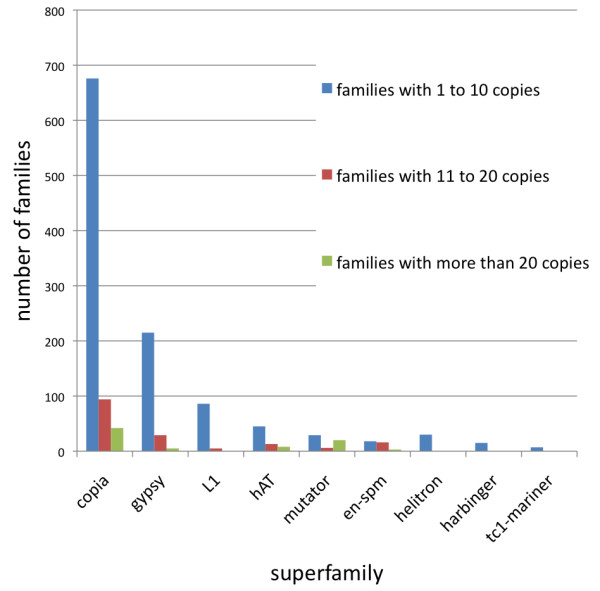
**Copy numbers in TE families within superfamilies.** Number of TE families in each one of the major superfamilies having 1–10, 11–20 or more than 20 copies in the genome.

We compared the TE sequences to EST databases to estimate the relative expression of each type of TE. The majority of ESTs queried were obtained from the same variety as was used for the WGS assembly [48]. To reduce redundancy, all of the putative TE sequences generated by the various *de novo* algorithms were first aligned to each other to generate clusters. Clustering was performed so that each member of a cluster had ≥80% similarity to every other member of the cluster. Each cluster was referred to as a family in accordance with the standards established by Wicker et al., [49]. A representative TE (usually the longest sequence) from each family was aligned to all available flax ESTs from Genbank. Table 2 shows the number and proportion of families in each major superfamily of TEs that aligned to ESTs. The LTR elements had the largest number of families that aligned to one or more ESTs. This was consistent with their observed abundance and coverage in the genome. LTR elements also had some of the highest proportions of expressed TE families. On the other hand, the *hAT* superfamily had fewer families than the LTR elements, but had a higher proportion of families with associated ESTs. To establish the copy number of each of the TE families, a representative sequence from each family (as described above) was aligned to the WGS assembly using BLAT. A threshold of 80% was used, following Wicker et al., [49]. Within superfamilies of TEs there were many families with only a few copies in the genome, and a small number of families with a high copy number (Figure 1). When the copy numbers within each family were compared to the relative number of ESTs aligned to that family, no correlation was observed (results not shown). This indicated that elements with a high copy number are not necessarily currently active. This was observed in all families, with the exception of the *hAT* superfamily, which showed a weak positive correlation (r = 0.66) between copy number and EST counts. 

**Table 2:** **Putative expression of *de-novo *identified families of TEs. TE families are constituted by sequences with similarity** ≥**80% among all its members**

**superfamily**	**total number of TE families**	**Number of families hitting at least one EST with a minimum coverage of 70% EST**	**proportion of TE families with putative expression**
*Copia*	819	77	9.40
*Gypsy*	263	20	7.60
*L1*	115	1	0.87
*hAT*	68	7	10.29
*Mutator*	60	0	0.00
*En-Spm*	38	1	2.63
*Helitron*	32	0	0.00
*Harbinger*	15	0	0.00
*Tc1-Mariner*	7	0	0.00

### Relationship of TEs to genes

**Figure 2:**
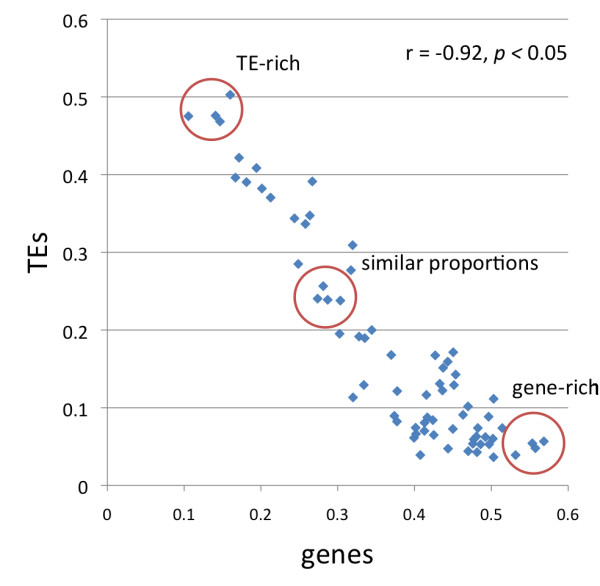
**Correlation of the proportional coverage between genes and TEs in scaffolds ≥ 1Mb.** Circled points indicate scaffolds selected for further analysis. TE-rich scaffolds (scaffolds 380, 50, 29 and 127); scaffolds with similar proportions of TEs and genes (scaffolds 33, 222, 151 and 132); gene-rich scaffolds (123, 464, 898 and 605).

**Figure 3:**
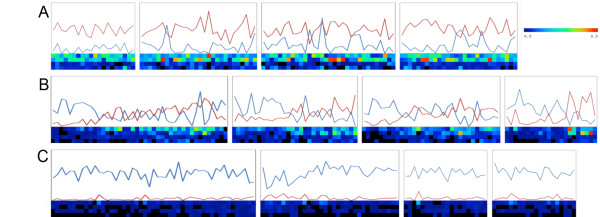
**Distribution of genes and transposable elements in representative scaffolds. A**) TE-rich scaffolds (scaffolds 380, 50, 29 and 127), **B**) Scaffolds with similar proportions of TEs and genes (scaffolds 33, 222, 151 and 132), **C**) Gene-rich scaffolds (123, 464, 898 and 605). The line graphs represent the proportional coverage (scale of 0.0 to 1.0) of TEs (red) and genes (blue) in 50kb windows. Each heat map row below each graph represent coverage of the four largest TE superfamilies – top to bottom: *Copia, Gypsy, L1, Mutator*, in 50kb windows (squares).

**Figure 4:**
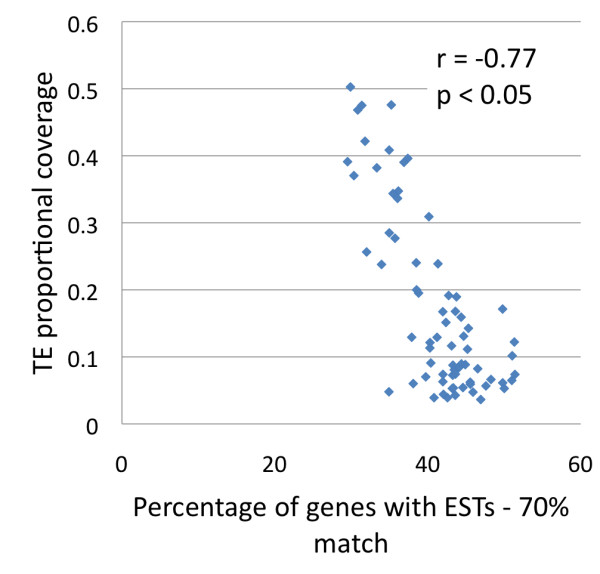
**Correlation between the percentage of genes with putative expression and TE proportional coverage.** The analysis was performed in scaffolds larger than 1 million bp. Each dot represent the x,y values of one scaffold. The TE proportional coverage is in a 0.0 to 1.0 scale.

**Figure 5:**
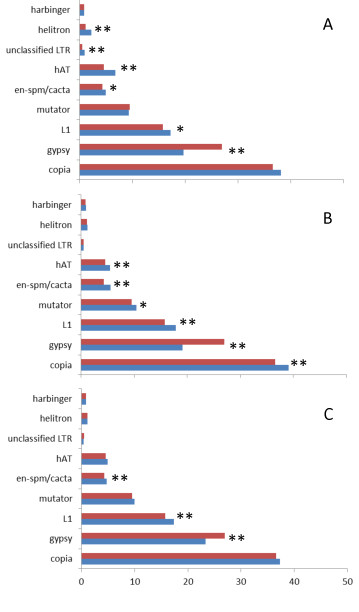
**Comparison of TE hits in and around genes to TEs in the genome. A**. In all scaffolds over 1 Mb vs. inside genes, **B**. In all scaffolds over 1 Mb vs. the adjacent 1kb up and downstream of genes, **C**. In all scaffolds over 1 Mb vs. the adjacent 5kb up and downstream of genes. The bars represent the percentage of the hits of each superfamily among all transposon hits in the scaffolds (red) or inside and in the adjacent gene regions (blue). Asterisks represent significance after Bonferroni correction at: *p < 5 x 10^-3 ^or **p < 5 x 10^-4^.

We investigated the distribution of TEs with respect to genes in the WGS scaffolds. We limited our analysis to 108,219,748 bp of genome assembly that was contained in 71 scaffolds of 1Mb or longer, since this allowed analyses of relationships of several contiguous genes and/or TEs. When individual scaffolds were analyzed as units, there was a highly correlated inverse relationship between the coverage of TEs and genes (r = −0.92, *p <* 0.05 – Figure 2) meaning that overall TE distribution in the flax genome was not completely random. The same trend was found when we analyzed each of the four most abundant superfamilies separately (*Copia, Gypsy, L1 and Mutator* – Additional file 2). The distribution patterns in the 71 studied scaffolds showed that some had dense coverage of TEs (and few genes), while others had many genes but few TEs, and still other scaffolds had similar proportional coverage of TEs and genes (Additional file 3). We chose 12 representative scaffolds to illustrate the trends of distribution. Four of these were gene-rich, four had a similar proportion of genes and TEs, and four had a higher proportion of TEs than genes (see Figure 2 for the selected 12 scaffolds). We divided the 12 scaffolds into equally sized 50 kb bins and then calculated the proportional coverage of genes, TEs and the four largest superfamilies of transposable elements (Figure 3). Within the scaffolds that were rich in TEs, we observed a few bins in which the frequency of genes was also high (blue line overlapping red line in Figure 3A). The TE rich scaffolds were dominated by *Copia* and *Gypsy* superfamilies, with the later having a higher proportion. The *L1* and *Mutator* elements had a lower proportional coverage than the LTR elements. The graphs did not show any apparent clustering pattern of any TE superfamily within each scaffold. A second group of scaffolds with similar proportional coverage of both TEs and genes showed several subregions in which TEs and genes overlapped or alternate in coverage (Figure 3B). Finally the gene-rich scaffolds seemed to be largely devoid of TEs (Figure 3C) and just a very few bins had proportional coverage of TEs close to 25%, while most bins were saturated for genes. We next used Monte Carlo (MC) statistics to test whether there was any scaffold (from our sample of 71 assembly units) in which TEs overlapped genes more frequently than expected by chance. Overlaps did not occur more frequently than expected by chance, whether the scaffolds were analyzed as individual units, or divided into 50 kb bins. Moreover, when the major superfamilies of TEs (*Copia, Gypsy,* the unclassified *LTR elements, L1, Mutator, En-spm/CACTA, hAT, Helitron and Harbinger*) were tested individually, none of the groups overlapped genes more than expected by chance in any scaffold (results not shown). Together this shows that that although regions of overlap did occur, and some TEs can be inserted into or close to genes, there were no individual scaffolds or bins with an unexpectedly high proportion of TEs in close association with genes. However, in scaffolds with a larger coverage of TEs, the probability of overlap with genes was higher as judged by a significant positive correlation between the proportion of bases overlapped by TEs in genes and the TE proportional coverage (r = 0.88 *p* < 0.05), and a significant negative correlation with the gene proportional coverage (r = −0.78 *p* < 0.05) (Additional file 4). In fact, when we used the 18,129 total predicted genes on these scaffolds to look for EST matches, it was found that the proportion of genes with matching ESTs in each scaffold was negatively correlated (r = −0.77 *p* < 0.05) with the proportional coverage of TEs (Figure 4). We next performed chi-square tests to determine whether any of the TE superfamilies, as compared to all other TEs, had a higher propensity to insert within or close to genes. Figure 5A shows that while the proportions were not significantly different (*p* = 0.005) for the *Copia*, *Mutator* and *Harbinger* elements, there were marginally significant differences for the *L1* and *CACTA* TEs, and strong significant differences for the remaining superfamilies. From this later group the *hATs, Helitrons* and the unclassified LTRs were found inside of genes more often than expected by chance, while *Gypsy* TEs were less commonly found inside genes than expected. When the analysis was repeated for the regions flanking the genes, three groups of DNA transposons (*hAT, CACTA* and *Mutator*) showed significantly higher affinity for the 1 kb of sequence that flanked genes when compared to their overall distributions (Figure 5B). The same was true for the retrotransposons *L1* and *Copia*, while *Gypsy* elements were significantly underrepresented in this region. Finally the analysis was repeated one last time for the flanking 5 kb of genes (Figure 5C) and in this opportunity only *CACTA, L1* and *Gypsy* showed significant differences with this later one still below the expected numbers on these gene flanking regions.

### Insertions of full LTR elements and evolution

**Figure 6:**
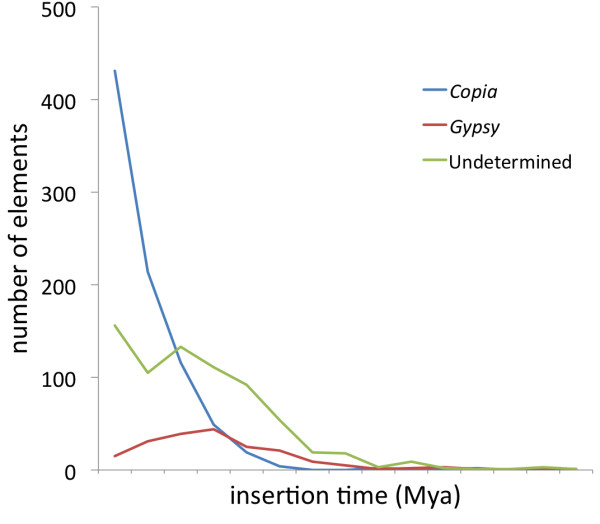
**Insertion age of LTR elements in millions of years (Mya).** Number of TEs inserted in the last 15 Mya for major groups of LTR retrotransposons.

**Figure 7:**
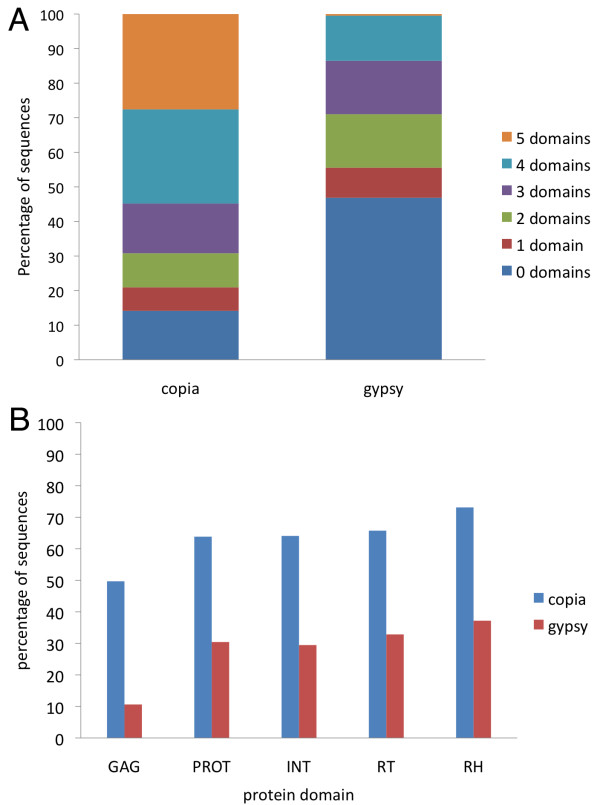
**Protein domains in LTR elements.** Proportion of LTR elements where the main protein domains (GAG, PROT, INT, RT, RH) were identified. In A, the number of domains that could be identified is shown. In B, the percentage of sequences of each superfamily that have each one of the domains is depicted.

A total of 2,266 putative LTR sequences found by LTR_finder and LTR_STRUC were filtered for redundancy, resulting in 1,767 unique sequences. After curation, some of these sequences were excluded from genome masking, because they contained internal non-TE genes, or other sequences of non-transposon origin, which would have resulted in masking of non-TE genome sections. However, the complete set of 1,767 unique sequences was used in subsequent analyses in order to more fully describe patterns of evolutionary importance like foreign DNA capture by TEs. Among the 1767 unique LTR sequences, 841 sequences corresponded to *Copia* elements, 207 were *Gypsy*, 667 were undetermined LTR elements, and the rest were LTRs that flanked other types of TEs. The *Copia* retrotransposons had an average size of 5.3 kb while the *Gypsy* TEs were 8.7 Kb on average. The LTR elements that had either regions of undetermined internal size, or regions bearing other types of TEs, were 5.9 kb on average (Additional file 5). When the LTR similarity was compared, the elements within these three groups had a similarity average of 95.4, 88.9 and 90.1 respectively; and the average distance between the TEs and the closest predicted gene was 3.2 kb (*Copia*), 7.6 (*Gypsy*) and 7.3 kb (undetermined) (Additional file 6). The divergence of intra-element LTR sequences was used to calculate the age of insertion of the unique elements, and a graph of their distribution in time was built (Figure 6). As seen in the figure, *Copia* elements had increasing activity in the last 5 million years, with many members active in the very recent past. There were 83 *Copia* sequences with 100% intra-element LTR similarity, and the average time of insertion of the elements in this superfamily was 1.4 Mya. In the meantime, the less abundant *Gypsy* elements increased activity around 7–8 Mya ago, but their activity started decreasing 3 to 4 Mya. There was only 1 *Gypsy* element with 100% LTR similarity and the average date of insertion for these elements was 4.1 Mya. Additionally there were four elements that were inserted more than 15 Mya (not included in Figure 6). Finally, the undetermined LTR elements (elements with internal regions not belonging to *Copia* or *Gypsy* domains) increased activity at around the same time as *Gypsy* elements, and were even more active than *Copia* elements until around 2.5 Mya, after which time they were still active but in a lesser proportion than *Copia*. There were 35 undetermined elements with 100% LTR similarity and their average date of insertion was 3.1 Mya. When the 119 retrotransposon sequences bearing 100% LTR pair intra-element similarity were mapped back to all scaffolds in the genome, we found a total of 147 insertion sites with 100% similarity to the original sequences. Only eight *Copia* TEs had more than one exact match (ranging from two to eight copies) in different genome regions (Additional file 7). When the match threshold was relaxed to 80% to find the number of copies that may have been related to the recently inserted copy, 19 elements had more than one related copy (ranging from 2 to 17 copies); from these, 18 were again *Copia* elements and one (with six copies) was an undetermined LTR element. When these recent insertion elements were compared to available ESTs, 19 *Copia* retrotransposons had a related EST (Additional file 8), and 11 undetermined elements had an EST match. Three of the undetermined elements had numerous EST matches (13, 16 and 92 hits respectively); but since the internal regions of these elements were either undetermined or matching sections of basal genes, the putative expression could correspond to genes located elsewhere in the genome. Finally, the distance between these recent TE insertions and genes showed that most of the recently inserted copies of LTR elements were located close to genes (Table 3). From among these LTR elements, all of those located within the first 1 kb flanking the genes were *Copia* when analyzing the 71 scaffolds of over 1 Mb, and 29 out of 31 were also *Copia* when analyzing all available scaffolds. To investigate the internal composition of the LTR sequences, all 1767 unique LTR sequences were used as queries in RepeatExplorer [50], which extracted protein domains of query sequences based on comparisons to a large database of transposons. Domains were identified only in the elements we had classified as *Copia* or *Gypsy*. All *Copia* and *Gypsy* elements matched domains only from their respective superfamilies with the exception of two TEs that we had classified as *Copia* elements but were found by RepeatExplorer to contain domains of *Gypsy* TEs. Whether these two instances represented nested insertions, recombination events, and chimeras or misassembled fragments is yet to be investigated. Overall, these results showed that our annotation of TEs was accurate (Additional file 9). Furthermore, *Copia* elements had more internal recognizable domains than *Gypsy* elements (Figure 7A). From a total of 841 complete LTR-*copia* elements, almost 55% contained four or five recognizable domains, while only 13% of the LTR-*gypsy* elements (a total of 207) had four recognizable domains, and only one sequence in which all five domains were recognized. When each domain was assessed separately, the proportion of *Copia* elements with a recognizable domain was always higher than in *Gypsy* (Figure 7B). These observations provide further evidence for a higher level of conservation and potential activity of *Copia* as compared to *Gypsy*. 

**Table 3:** Frequency of recent LTR elements insertions in proximity to predicted genes

**distance from the closest gene**	**scaffolds over 1MB**		**all scaffolds**	
	**number of LTR elements**	**proportion of the number of LTR elements**	**number of LTR elements**	**proportion of the number of LTR elements**
0-1000	13	46.43	31	43.06
1001-2000	4	14.29	12	16.67
2001-3000	2	7.14	5	6.94
3001-4000	1	3.57	4	5.56
4001-5000	1	3.57	2	2.78
>5000	7	25.00	18	25.00

## Discussion

### TEs in the flax genome

The flax genome is estimated to be 373Mb in length, and we have reported here and previously that at least 23% of its sequence is made of TEs [40]. We expect that the actual TE coverage of the complete genome is higher than the 23% we reported, for the following reasons: (i) unclassified repeated sequences found by our *de novo* approach could constitute new or highly divergent types of TEs, but these were not used for masking; (ii) numerous LTR elements with unknown or non-TE internal domains were not included in the masking, and we did not use specific algorithms to identify possible TEs that lacked internal recognized domains; (iii) the WGS assembly may be missing some regions that are rich in repeated sequences [40]. If the complete genome sequence could be analyzed, including regions missing from the WGS assembly, we expect not only that the proportion of TEs would increase, but also the relative abundance of the main superfamilies could change since *Gypsy* elements are rich in heterochromatic regions [51-57], which are usually more difficult to assemble. Nevertheless, our estimate of genome coverage by TEs is comparable to what has been found for other sequenced plant genomes with sizes slightly larger than flax (e.g. *Oryza sativa* - 35% TEs, *Lotus japonicus* - 30.8%, *Medicago truncatula* 38%) [9]; indeed it has been proposed that in angiosperms, approximately one third of the genome is made up of TEs [9], which is in general agreement with our estimate for flax. Although TE content may be more related to genome size variation in plants with larger genomes [58] there is a trend showing that genome size correlates positively with the abundance and expansion of TEs [9]. While there are exceptions to this rule, flax with its smaller genome has a much lower percentage of TEs when compared to larger genomes like maize with over 85% TEs [11]. We found that LTR elements (especially *Copia*) dominated the population of TEs in the flax genome (Table 1). LTR retrotransposon abundance has been described in numerous plant species including some of the closely related species to flax that have been fully sequenced. In castor bean (*Ricinus communis*) the length covered by LTR elements accounts for about one third of all repeats while DNA TEs constitute less than 2% [59]; while in poplar (*Populus trichocarpa*) LTR elements constitute around 17% of the bases of all repeats (including low complexity repeats), and DNA TE content is close to 5% [60]. Although the proportion of sequence covered by LTR elements in flax is larger than in castor or poplar, the predominance of LTR elements is typical in many plant genomes [see supplementary table 7 in [61]. However, in most characterized genomes it is the *Gypsy* group that outnumbers the *Copia* group (Additional file 10). *Ty3-gypsy* elements are dominant in: *Brachypodium distachyon *[62], *Oryza sativa *[63], *Zea mays *[11], *Sorghum bicolor *[64], *Carica papaya *[65], *Arabidopsis thaliana *[10]- [LTR element coverage obtained from 61], *Fragaria vesca *[66], *Malus domestica *[61], *Glycine max *[67], *Phaseolus vulgaris* (data obtained from Phytozome - http://www.phytozome.net/)*, Populus trichocarpa *[60] - [LTR element coverage obtained from 61] *and Ricinus communis *[59]*.* Only *Linum usitatissimum* (this study), *Vitis vinifera *[68]*, Theobroma cacao *[69] and *Cucumis sativus *[70] seem to have higher coverage by the *Copia* superfamily, although in these last two genomes only the number of elements and not the coverage in bp was shown in the referenced papers and therefore they could not be included in Additional file 10. The prevalence of a superfamily may be related to amplification events of specific groups of TEs and to the activity of such elements, which may depend on just a few active copies of the family [12]. An interesting example is the genus *Gossypium*[71], in which one of the species with the smallest genomes had a high density of *Copia* elements. *Gossypium* species with larger genomes had an increased copy number of *Gypsy* elements, most of which represented just one subgroup of the *Gypsy* sequences. Such amplification can be lineage-specific and therefore result in changes in genome size [71-73]. In flax we found that *Copia* elements were abundant, diverse and some members were recently active (see below), which would explain a higher current influence of such elements. LINEs and *Mutator* elements were the most abundant after the LTR retrotransposons (Table 1). Although these two types of elements seem to be or have been fairly active, their lesser abundance when compared to LTR elements can be explained at least in part by their transposition mechanisms. For example, the mechanism of non-LTR retrotransposition generally creates truncated copies of the elements, which would largely decrease their coverage in a genome [74]; additionally plant LINEs are very diverse and heterogeneous due to the error-prone mechanism of their reverse transcriptase, and the accumulation of mutations during long evolutionary periods [74], which limits their identification. In the case of the *Mutator* elements, their cut and paste transposition does not increase copy number as much as retrotransposition. Additionally, non-autonomous gene-carrying *Mutators* or MULEs (Mu-like elements) can sometimes be difficult to identify by traditional bioinformatics approaches, and seem to be widely divergent [75]. Thus, in flax, identification of such elements may also be influenced by the high mutation rates and transposition mechanisms, resulting in lower percentages of identified *Mutator* and LINE elements.

### Putative expression and abundance of main families of TEs

Besides being abundant, LTR elements were also diverse in the flax WGS assembly. The number of families (Table 2) was probably overestimated, since as a result of the masking process, some of the fragments we found may in fact be different segments of a single element. Nevertheless, there was a general correlation between superfamily genome coverage and the number of families found. Alignment of TEs to EST databases showed that just a small proportion of flax TEs might be active, most of which were *Copia* LTR elements (Table 2). Our results are in agreement with the survey done in over 200,000 ESTs for sugarcane where *Copia* elements had more matching ESTs than *Gypsy* retrotransposons [76], although in sugarcane (but not flax), DNA transposons also seemed to be fairly active. In plants, TE activity depends on regulatory factors including stress-driven transcriptional regulation and epigenetic silencing, which allow activation of just a few elements under specific environmental and developmental circumstances [12,37]. For example in maize, where more than 80% of the genome is made of TEs, a survey of over 2 million ESTs showed that only 1.5% of them matched TEs, and most of the families with putative activity were LTR retroelements. Thus for flax, as well as for most plant species studied, the activity of TEs seems relatively low, and may increase to detectable levels only in response to stress. Additionally, it has been shown that in certain families of TEs the percentage of polyadenylated expressed sequences is low [77]. Because most EST libraries are built by poly-A extension, this may artificially limit the proportion of expressed TEs that can be detected by alignment to ESTs. We also found that across all TEs there were fewer families with high copy numbers throughout the genome and most families within each superfamily had less than 10 copies (Figure 1), which is in agreement with findings in soybean where 78% of LTR families are present at copy numbers below 10 [67]. While low copy number could be related to low transposition rates, mechanisms like high mutation rate [78], recombination [79] and nested insertions [80], create rapid variability in TEs that results in divergence among TEs, and therefore, a low number of similar sequences. Since we did not find a correlation between copy numbers and putative expression (related ESTs), it is more likely that mechanisms of divergence and not the transposition of low copy number families account for the trend we found. This lack of correlation is in agreement with previous findings in maize [36] and contradicts a previous view that low copy number elements are the ones that are predominantly expressed [81].

### Relationship of TEs to genes

The location of TEs in the flax genome was not completely random. It was evident that some scaffolds came from genomic regions rich in TEs (especially retrotransposons, which constituted the bulk of flax TEs – Figure 3) and were highly depleted in genes. Conversely, other scaffolds were rich in genes but depleted in TEs, and still others had similar coverage of TEs and genes. A global negative correlation of TE coverage and gene coverage agrees with a model in where there is purifying selection against TEs in coding regions to avoid detrimental effects on genome function; this model was clearly presented for Arabidopsis [82]. In sequenced genomes such as those of *Sorghum bicolor *[64] or *Brachypodium distachyon *[62], where the distribution of TEs has been mapped to chromosomes, the bulk of retrotransposons seem to be clustered around the centromeres, while less are close to gene-rich regions probably due to rapid elimination by controlling and selective host mechanisms [64]. When each of the 71 largest scaffolds was analyzed as an individual unit, there was no evidence for an overall pattern in which TEs occurred inside genes more that expected by chance (Additional file 4). However, there were certain superfamilies that were more likely to do so when compared to the rest of TEs (Figure 5). Several DNA TE superfamilies and L1 elements fell inside and close to genes more often than expected, while *Gypsy* elements were always underrepresented in and around genes, and *Copia* retrotransposons were only significant in the first 1 kb flanking genes. In Arabidopsis an analysis to find chimeric genes/TEs showed significant differences for *Copia, En-Spm, Gypsy* and *Helitrons *[35]. While both for flax and Arabidopsis there was an overrepresentation of *En-Spm* and underrepresentation of *Gypsy* TEs, *Copia* elements were overrepresented inside genes in Arabidopsis but not in flax, and *Helitrons* were underrepresented in Arabidopsis while this superfamily and *hATs* were significantly overrepresented in flax. The overrepresentation of class II TEs in flax genes is consistent with reviews describing the close association of genes and these elements including the domestication of transposon proteins into genes [4,83]. For example, TEs like *En-Spm/CACTA* are closely associated with genes in the *Triticaceae* and they may even capture gene fragments as they move and recombine in the genome [84]. In the case of *Helitrons*, extensive gene capture and shuffling mediated by these elements has been reported [85-88]. For *hATs*, gene shuffling has been reported in maize [89], and experiments with rice have shown that the *nDart1* and its relatives belonging to *hATs* tend to fall within of very close to genes [90]. In the meantime, while *Mutator* elements were not overrepresented inside genes, they were abundant in the 1 kb of DNA flanking them. The close relationship of *Mutator* elements with genes allow TE-mediated gene movement, as has been shown for *Mutator*-like elements (MULEs) in rice [19] and Arabidopsis [20], and relates to fixation of TE enzymes like transposase, which is part of *FHY3* and *FAR1* genes involved in phytochrome A signalling [21,22]. Putative homologs of these two transposase bearing genes were also found in flax (result not shown). The *Gypsy* underrepresentation in gene coding regions of flax could be related to their tendency to cluster close to centromeric regions. This has been shown in grass species [52], in plants like sunflower [51,55,56] and has more recently been proposed for plants like poplar [57] and Arabidopsis [91]. It has been speculated that the reason for this insertion bias may be related to a specific domain in the integrase protein [91-93]; and such differences in integrase proteins may also be related to the differing distributions between *Gypsy* and *Copia* elements. Finally, *Copia* elements were overrepresented in the 1 kb of sequence that flanked genes (Figure 5). In Arabidopsis, the random pattern of *Copia* insertion allows them to insert close to coding regions [91], although in time, the elements are subjected to negative selection. A similar pattern could be true for flax since we found that many recently inserted *Copia* TEs were close to genes (Table 3). This insertion pattern might have important implications since TEs close to genes can become positive regulators of gene expression via their *cis-*acting elements (in LTRs) or may become targets for epigenetic silencing, which would affect the adjacent gene regions [94-96]. To test if there was a general pattern of regulation of genes by TEs, we matched available ESTs to the predicted genes of the flax genome (Figure 4). The negative correlation of TE coverage and gene expression found means that genes in regions that are rich in TEs could be affected by their nearby insertion. It is likely that genes in close proximity of TEs are affected negatively, because most often these regions are targeted for heterochromatization (and silencing) [96-98] and TE insertion can also cause disruption of genes.

### Insertions of full LTR elements

Most of the non-redundant elements with two identifiable LTRs belonged to the *Copia* superfamily, but a large proportion of retroelements had non-identifiable internal regions, or regions that corresponded to host genes or other non-LTR TEs as has also been shown for poplar (Additional file 5) [57]. Many of these may constitute either non-autonomous elements, or genes captured by TEs. As it turns out, these two concepts are not mutually exclusive. For example, in soybean (*Glycine max*), an element has been described with an insertion of 10.5 kb containing a mixture of segments derived from non-coding sequence and disease resistance genes [99]. These elements could still be actively driven by autonomous elements if they conserve their LTRs, polypurine tracts (PPTs), and primer binding sites (PBS’). Many of the undetermined elements in flax had such features (these are part of the recognition algorithm of LTR_STRUC and LTR_FINDER), and therefore may still be active. In fact 32 of these undetermined elements had 100% LTR pair similarity and 69 had at least 99% similarity, meaning these TEs constitute relatively recent insertions. It is likely that at least some of the larger flax LTR elements could be classified as LARDs (Large Retrotransposon Derivatives) which have been characterized in detail in the *Triticaceae,* rice and *Medicago *[100-102], while some of the shorter than expected LTR retrotransposons are probably TEs that have lost their internal coding regions and are usually classified as Terminal-repeat Retrotransposons in Miniature (TRIMs) [103]. In terms of TE sizes, calculated estimates for plants range from 2–11.8 kb for *Copia* elements, and from 4.6-18 kb for *Gypsy* elements [104]. However, a survey of LTR retroelements in rice using LTR_finder found large variation ranges in LTR retrotransposons [105] which is in agreement with the larger variations found in flax (Additional file 5). Nevertheless our averages agree with the *Gypsy* elements having larger sizes *Copia* as is common in most plants. When comparing the activity of the LTR elements (Figure 6), the *Copia* elements appeared to be increasingly and continuously active in the last 5 million years. In the meantime *Gypsy* elements have been active for the last 7–8 million years but to a lesser extent than *Copia* and the undetermined elements. In fact, after a peak of activity 3–4 Mya, *Gypsy* elements have been less active until the present. In comparison, for poplar, the activity of *Copia* full length TEs does not seem to overshadow the activity of *Gypsy* elements, but full *Copia* elements are more abundant than *Gypsy*[57]. Although the activity of all these retroelements varies, it is interesting to notice that between 5 to 10 Mya, all of them may have been triggered. It is tempting to speculate that a duplication event of the flax genome may have triggered activity of the retrotransposons, and indeed whole genome duplication in this time frame has been inferred based on molecular phylogenies and analysis of Ks distribution in protein coding genes [38,40]. However, rapid turnover of elements is also common [106,107] and could account for the absence of detection in more ancient evolutionary times since TEs may become unrecognizable. When evaluating only the most recent flax LTR element insertions, it was shown that *Copia* LTR elements have more copies, putative expression and are located close to genes. The lone, recently inserted *Gypsy* element had no related ESTs. A similar insertion pattern was seen in Arabidopsis where the number of *Copia* elements with identical LTRs is higher than in *Gypsy* elements, and recent *Copia* insertions are closer to genes than *Gypsy *[91]. It can not be ruled out that the short read assembly methodology used for the flax WGS [40] is biased towards more efficient identification of regions surrounding genes. Nevertheless we found that *Gypsy* elements followed the opposite trend of *Copia*, meaning that both types of elements were detected, whether they were closely associated with genes or not. This observation and the agreement with other studies on this trend [91] supports our conclusions.

## Conclusions

We showed that transposable elements in flax occupied more than 23% of the flax WGS assembly and were dominated by LTR elements. The distribution of TEs was not random and there were genomic regions that were enriched by these repetitive sequences, which may constitute heterochromatic sections of the genome. In regions shared by both TEs and genes, transposons may have a repressive effect on gene expression as demonstrated by a negative correlation between TE coverage and gene expression. Overrepresented families in close proximity or overlapping genes were mainly from the DNA transposon group, but the *Copia* group was also often localized to the flanking regions of genes. *Copia* retrotransposons have been increasingly active in the last 5 million years and have more members with conserved internal domains that contrast with a lower activity and conservation of *Gypsy* elements. It is possible, however, that older insertions are more difficult to tag by the high rate of mutations especially for TEs located to heterochromatic regions. Because of their recent activity, abundance and diversity, the *Copia* elements are potential shapers of the flax genome. Further studies, especially under stress-eliciting conditions, are necessary to understand the regulatory effect on adjacent genes and how their activation patterns may have influenced evolution of other flax species.

## Methods

### Identification of putative TEs within the flax WGS assembly

An unmasked WGS assembly of flax comprising 318,250,901 bases was used as input for TE detection [40]. *De-novo* identification of transposable elements was performed using RepeatScout [108], PILER [109], LTR_finder [110] and LTR_STRUC [111]. Repeats identified by RepeatScout, under default parameters, were filtered for low complexity using Tandem Repeats Finder [112] and nseg [113]. Repeats with less than 10 hits in the genome were eliminated from the library. For PILER-DF [109] analysis the full genome was compared to itself using PALS (part of the PILER implementation) using the default parameters. Families of dispersed repeats were created using a minimum family size of 3 members and a maximum length difference of 5% between all family members. The consensus sequence for each family was created after aligning the sequences with MUSCLE [114]. LTR TEs were found using LTR_finder using the option –w 2 to get a table output that could be parsed to obtain the sequences corresponding to the elements. LTR_STRUC was used under default parameters. The sequences output by all of these programs were used to create a unified repeat library that could be compared to previously characterized elements. Annotation of the repeats was performed comparing the library to a *Viridiplantae* TEs database downloaded from Repbase (http://www.girinst.org/repbase/ - update 20110920) and a Plant Repeat Database (http://plantrepeats.plantbiology.msu.edu/) of TEs created from the families Brassicaceae, Fabaceae, Gramineae and Solanaceae (v2_1_0 update 20112006), using tBLASTx and BLASTn, and to the RepeatPeps database of TEs that comes with RepeatMasker (update 20110920) using BLASTx. To test whether TEs might have captured fragments of other genes or belong to gene families instead of TE families, BLASTx was performed against the Genbank nr database. Repeats were classified in a TE superfamily [49] if they showed E values of at least 1e-5 with a common annotation in at least two of the databases to which they were compared. Repeats characterized as putative TEs by the previous approach were joined to the *Viridiplantae* database of TEs (update 20110920) to use as a library for comparison to find the distribution and coverage TEs in the genome assembly using RepeatMasker v-3.3.0 (http://www.repeatmasker.org/). RMBlast was used as search algorithm with Smith-Waterman cutoff of 225 (this cutoff was used for all RepeatMasker analyses). To automatically annotate the masked regions (matches of the TEs in the genome) in their respective TE superfamilies a custom Perl script was used (kindly provided by Robert Hubley - Institute for Systems Biology, http://www.systemsbiology.org/ -). A table for TEs abundance and coverage was built after filtering and annotation. The percentages were calculated for the elements based on the total number of bases including runs of Xs and Ns since some elements can also include at times undetermined bases; therefore total percentages may differ a slightly from those reported in the original description of the flax genome [40]. The TE values of the WGS assembly were compared to BAC-end sequencing TEs [47].

### Putative expression and distribution of TE families

Clusters of TEs with 80% similarity within each superfamily were created using CD-HIT [115]. Only *de-novo* identified members were used for this analysis since they represented TE sequences identified from the flax genome. The members of each cluster were said to represent a family of TEs, according to the terminology presented by Wicker et al., [49]. One representative member of each family (longest sequence in each cluster) was used for comparison against 286,252 flax ESTs from Genbank using BLAT [116]. A hit to an EST was classified as positive only if 70% of the EST sequence matched to the query sequence. A family was said to be putatively expressed if it had at least one EST match. The proportion of TE families with expression was calculated for each of the major groups. The same analysis was done comparing the TE family representative sequences to the flax assembly, and a TE was considered as a representative copy of the TE family if it matched in 80% of its sequence to the query. A coefficient of correlation was established between copy number in each family and ESTs matches.

### Relationship of TEs to genes

The distribution of TEs relative to predicted genes in the WGS assembly was analyzed for all scaffolds ≥ 1 Mb (71 large scaffolds). The proportional coverage and the statistics applied for both genes and TEs were obtained for both: full scaffolds and windows of 50 kb within each scaffold after mapping the coordinates of predicted genes and TEs to the scaffolds using the Genomic Hyperbrowser [117] (http://hyperbrowser.uio.no/rc/ - candidate version). To test whether the distribution of TEs and genes was correlated, a correlation coefficient was calculated for the proportional coverage of the large scaffolds. Proportional coverage graphs and heat maps for comparison of TEs and genes were built for selected scaffolds divided in 50 kb window units or bins (four scaffolds with large proportions of TEs, four with large proportion of genes and four with similar proportions of TEs and genes). The heat maps were built using with Multi Experiment Viewer [118]. To test whether TEs overlapped genes more than expected by chance in any of the scaffolds over 1 Mb we used Monte Carlo (MC) methods [119], preserving the segment lengths and position of the genes and changing the positions of the TEs to create the random probability, with a minimum of 100 MC samples and unlimited maximum number, a sequential MC threshold of 20 and a MCFDR of 0.05; the analysis was repeated for the scaffolds divided into 50 kb bins (2182 bins in total). For generating the random samples the lengths of genes and TEs were conserved, and only the TE positions were randomized which closely reflects the biological context. The proportion of gene coverage overlapping TE sections was calculated from the total base pairs covered by genes in each scaffold and the total base pairs calculated as being overlapped both by genes and TEs. Scaffolds having high proportion of overlap were further analyzed by calculating the overlap proportion in 50 kb bins. The MC statistical analysis was repeated using TE superfamilies. Putative expression of the genes in the scaffolds over 1Mb was determined by comparing the predicted mRNAs to 286,252 ESTs of flax from Genbank using BLAT [116]. A hit to an EST was classified as positive only if it matched 70% of the EST sequence. A gene was said to be putatively expressed if it had at least one EST match. The proportion of genes with expression was calculated for each one of the large scaffolds, and compared with the proportional coverage of TEs. To find out if any of the superfamilies had a bias to insert within genes when compared to the other superfamilies, the number of TE hits inside genes of each superfamily was determined with the Genomic Hyperbrowser [117] using the middle point of the TE sequences to determine if the TE was inside the gene. Then the number of TE hits inside genes was compared to the number of hits in all the scaffolds over 1 Mb using heterogeneity chi-square tests and a Bonferroni correction [120]. These analyses were then repeated to compare the TEs in the adjacent 1 kb, and in the adjacent 5 kb (upstream or downstream from the genes).

### Insertions of full LTR elements and evolution

Since LTRs were the most prevalent elements in the flax genome, they were analyzed in further detail. Results from LTR_finder and LTR_STRUC were filtered for redundant sequences using CD-HIT [115]. Since at the time of insertion both LTRs from LTR retrotransposons are 100% similar, the divergence between LTR pairs in every putative element can be used to determine the age of the elements. We used ClustalW [121] for aligning LTR pairs and used the Kimura two parameter method [122] to estimate the nucleotide substitution (*K*). To estimate the age of insertion we used the following equation: *t = K/2r,* where *t* corresponds to the insertion time in millions of years, *K* corresponds to the number of nucleotide substitutions per site and *r* corresponds to the nucleotide substitution rate. In this case we chose a rate of 1.5 X 10^-8^ as reported for chalcone synthase and alcohol dehydrogenase genes in *Arabidopsis* and *Arabis* species; this rate has been previously used for dating LTR retrotransposon insertions in Arabidopsis [91], and it is very close to the estimate used for dating LTR retroelements in rice [123] which assumes at least a 2-fold higher mutation rate in TEs than in coding regions. The library of non-redundant elements with 100% LTR similarity was used to search the flax assembly and the flax ESTs using BLAT [116] to establish the abundance, distribution and overall putative expression of the recent insertions. Only hits that covered 100% of the query sequence were selected (no gaps or miss-matches), as these represented complete elements mapped to the genome. The segment distances between LTR retrotransposons elements and the closest genes were determined using the Genomic Hyperbrowser [117]. Finally, non-redundant LTR element sequences were used as input to extract protein domains from both *Copia* and *Gypsy* elements using RepeatExplorer by comparing flax LTR elements to a database of curated LTR retrotransposon domain sequences; the parameters for comparison were: minimum similarity 60%, minimum identity 40% and the proportion of the hit length from the length of the database sequence was set to 0.8 [50]. The domains were tabulated to discover the distribution of conserved domains in each superfamily.

### Availability of supporting data

The data sets supporting the results of this article are included within the article (Additional files 1, 2, 3, 4, 5, 6, 7, 8, 9, 10).

## Competing interests

The authors declare that they have no competing interests.

## Authors' contributions

LGG conducted all analyses and wrote the manuscript. MKD supervised the research and edited the final version of the manuscript. All authors read and approved the final manuscript.

## Authors' information

LGG: Department of Biological Sciences, University of Alberta, Edmonton, AB Canada T6G 2E9. Centennial Centre for Interdisciplinary Science (CCIS), 5–114.MKD: Department of Biological Sciences, University of Alberta, Edmonton, AB Canada T6G 2E9. Centennial Centre for Interdisciplinary Science (CCIS), 5–114.

## Supplementary Material

Additional file 1 **Annotation of *de novo *repeats.** Annotation of *de-novo* repeats. Click here for file

Additional file 2 **Correlation between TE and gene coverage on scaffolds ≥ 1Mb.** Scatter plots of correlation of the proportion of coverage between genes and the four largest transposable elements (TEs) superfamilies in scaffolds larger than 1 million bp. Click here for file

Additional file 3 **Proportional coverage of genes and TEs.** Proportional coverage of genes and TEs in scaffolds over 1 Mb. Click here for file

Additional file 4 **Monte Carlo tests.** Monte Carlo test for probability that TEs are overlapping genes more than expected by chance. Click here for file

Additional file 5 **Filtered LTR elements.** Filtered LTR elements. Click here for file

Additional file 6 **Distances of LTR elements to their closest gene.** Distances of LTR elements to their closest gene. Click here for file

Additional file 7 **BLAT analysis of recent LTR TE insertions in genome.** BLAT analysis of recent LTR TE insertions in genome. Click here for file

Additional file 8 **BLAT analysis of recent LTR TEs against ESTs.** BLAT analysis of recent LTR TEs against ESTs. Click here for file

Additional file 9 **Protein domains in LTR elements.** Identification of protein domains in LTR elements. The name of the sequences are made using superfamily, algorithm, coordinates and similarity percentage between LTR pairs. Click here for file

Additional file 10 **Percentage of bp covered by LTR retrotransposon superfamilies.** Percentage of bp covered by LTR retrotransposon superfamilies in characterized genomes. Plant species are organized according to phylogenetic relationships. The figures for each genome correspond to *Brachypodium distachyon *[62], *Oryza sativa *[63], *Zea mays *[11], *Sorghum bicolor *[64], *Vitis vinifera *[68]*, Carica papaya *[65], *Arabidopsis thaliana *[10]- [LTR element coverage obtained from 61], *Fragaria vesca *[66], *Malus domestica *[61], *Glycine max *[67], *Phaseolus vulgaris* (data obtained from phytozome - http://www.phytozome.net/)*, Populus trichocarpa *[60] - [LTR element coverage obtained from 61], *Linum usitatissimum* (flax - this study), *Ricinus communis *[59]*.* The transposable elements from the genomes of *Theobroma cacao *[69] and *Cucumis sativus *[70] have more *Copia* than *Gypsy* elements but could not be included in the figure since their actual coverage on the genome was not specified. Click here for file
