# Supplementary material for: Identification, characterization and distribution of transposable elements in the flax (Linum usitatissimum L.) genome
Source: BMC Genomics. 2012 Nov 21;13:644. doi: 10.1186/1471-2164-13-644 (PMC3544724; doi:10.1186/1471-2164-13-644)
Supplement: Additional file 2 — Correlation between TE and gene coverage on scaffolds ≥ 1Mb. Scatter plots of correlation of the proportion of coverage between genes and the four largest transposable elements (TEs) superfamilies in scaffolds larger than 1 million bp. [file 1471-2164-13-644-S2.docx]

**Additional file 2. Scatter plots of correlation of the proportion of coverage between genes and the four largest transposable elements (TEs) superfamilies in scaffolds larger than 1 million bp.**
