# Supplementary material for: Identification, characterization and distribution of transposable elements in the flax (Linum usitatissimum L.) genome
Source: BMC Genomics. 2012 Nov 21;13:644. doi: 10.1186/1471-2164-13-644 (PMC3544724; doi:10.1186/1471-2164-13-644)

**Additional file 10. Percetage of bp covered by LTR retrotransposon superfamilies in characterized genomes. Plant species are organized according to phylogenetic relationships. The figures for each genome correspond to *Bracypodium distachyon* [9], *Oryza sativa* [12], *Zea Mays* [11], *Sorghum bicolor* [10], *Vitis vinifera* [20]*, Carica papaya* [14], *Arabidopsis thaliana* [13]- [LTR element coverage obtained from 16], *Fragaria vesca* [65], *Malus domestica* [16], *Glycine max* [15], *Phaseolus vulgaris* (data obtained from phytozome -** [**http://www.phytozome.net/**](http://www.phytozome.net/)**)*, Populus trichicarpa* [21] - [LTR element coverage obtained from 16], *Linum usitatisimmum* (flax - this study),  *Ricinus communis* [17]*.* The transposable elements from the genomes of *Theobroma cacao* [7] and *Cucumis sativus* [19] have more *Copia*  than *Gypsy* elements but could not be included in the figure since their actual coverage on the genome was not specified.**


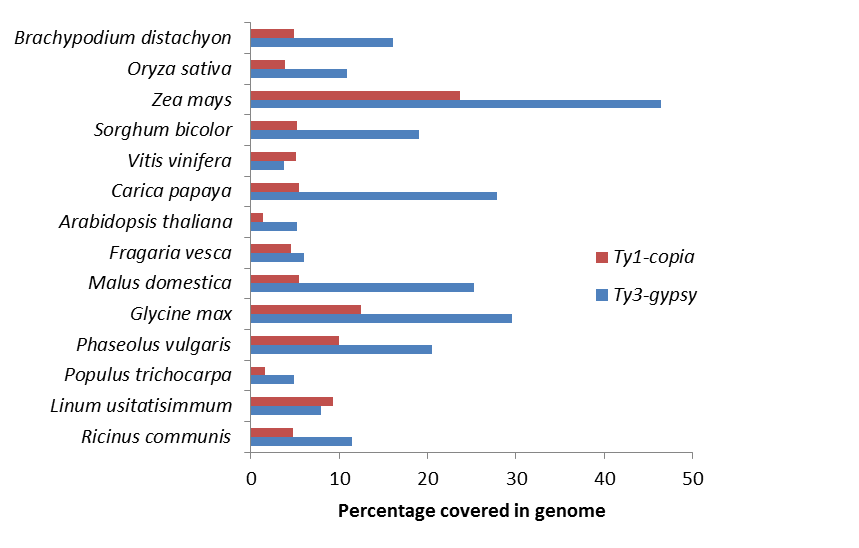

Supplement: Additional file 10 — Percentage of bp covered by LTR retrotransposon superfamilies. Percentage of bp covered by LTR retrotransposon superfamilies in characterized genomes. Plant species are organized according to phylogenetic relationships. The figures for each genome correspond to Brachypodium distachyon [62], Oryza sativa [63], Zea mays [11], Sorghum bicolor [64], Vitis vinifera [68], Carica papaya [65], Arabidopsis thaliana [10]- [LTR element coverage obtained from 61], Fragaria vesca [66], Malus domestica [61], Glycine max [67], Phaseolus vulgaris (data obtained from phytozome - http://www.phytozome.net/), Populus trichocarpa [60] - [LTR element coverage obtained from 61], Linum usitatissimum (flax - this study), Ricinus communis [59]. The transposable elements from the genomes of Theobroma cacao [69] and Cucumis sativus [70] have more Copia than Gypsy elements but could not be included in the figure since their actual coverage on the genome was not specified. [file 1471-2164-13-644-S10.docx]
